# Associations between socioeconomic position and asthma: findings from a historical cohort

**DOI:** 10.1007/s10654-012-9703-9

**Published:** 2012-06-14

**Authors:** Sumaiya Patel, John Henderson, Mona Jeffreys, George Davey Smith, Bruna Galobardes

**Affiliations:** 1 MRC Causal Analyses in Translational Epidemiology Centre, School of Social and Community Medicine, University of Bristol, Bristol, UK; 2 School of Social and Community Medicine, University of Bristol, Bristol, UK

**Keywords:** Asthma, Socioeconomic position, Early life, Atopy

## Abstract

Understanding the association between asthma and socioeconomic position (SEP) is key to identify preventable exposures to prevent inequalities and lessen overall disease burden. We aim to assess the variation in asthma across SEP groups in a historical cohort before the rise in asthma prevalence. Male students participating in a health survey at Glasgow University from 1948 to 1968 (n = 11,274) completed medical history of bronchitis, asthma, hay fever, eczema/urticaria, and reported father’s occupation. A subsample responded to postal follow-up in adulthood (n = 4,101) that collected data on respiratory diseases, early life and adult SEP. Lower father’s occupational class was associated with higher odds of asthma only (asthma without eczema/urticaria or hay fever) (trend adjusted multinomial odds ratio (aMOR) = 1.23, 95 % CI 1.03–1.47) but with lower odds of asthma with atopy (asthma with eczema/urticaria or hay fever) (trend aMOR = 0.66, 95 % CI 0.52–0.83) and atopy alone (trend aMOR = 0.84, 95 % CI 0.75–0.93). Household amenities (<3), in early life was associated with higher odds of adult-onset asthma (onset > 30 years) (OR = 1.48, 95 % CI 1.07–2.05) though this association attenuated after adjusting for age. Adult SEP (household crowding, occupation, income and car ownership) was not associated with adult-onset asthma. Lower father’s occupational class in early life was associated with higher odds of asthma alone but lower odds of asthma with atopy in a cohort that preceded the 1960s rise in asthma prevalence. Different environmental exposures and/or disease awareness may explain this opposed socioeconomic patterning, but it is important to highlight that such patterning was already present before rises in the prevalence of asthma and atopy.

## Introduction

Asthma is amongst the most common chronic diseases in childhood and is a large source of patient morbidity [1, 2]. The prevalence of asthma has increased considerably over the last 50 years [2]. This rise in prevalence has occurred rapidly over a relatively short period of time, which indicates that this is not due to genetic shifts but most likely due to alterations in lifestyle and/or environment. There have been a number of environmental changes that have occurred in parallel with the increase in these conditions. These include changes in diet [3], in allergen exposure [4], in microbial exposure [5], pollution [6], smoking [7], antibiotic use [8] and obesity [9]. However, to date, no single environmental exposure has been shown to explain the rising trend.

In addition to changes over time, there is substantial variation in the prevalence of asthma across countries and socioeconomic conditions within a country. Data from the US [10] and Europe [11] suggests that currently prevalence of asthma is higher with lower socioeconomic position (SEP), whilst allergic sensitisation [12] and hay fever [13] demonstrate lower prevalence with lower SEP. Characterising the association between SEP, asthma and atopic conditions is important in order to quantify an inequitable burden in subgroups of the population. In addition, understanding the factors that give rise to these inequalities can help to identify potential causal exposures.

A study from Sweden shows that the socioeconomic pattern has changed over time and that changes differ whether atopic or non-atopic asthma is considered [14]. A faster rise of asthma with allergic rhinitis resulted in this condition being more prevalent among the lower educated groups born early to mid 1970s [14]. It had previously been a condition associated with higher educated groups. Understanding the underlying mechanisms behind these changes is key to identify preventable exposures. It is not clear whether a similar scenario has taken place in the UK. Few studies have reported the socioeconomic distribution of asthma prior to the rises in prevalence in the UK and only one has been able to include information on atopy [14]. The Glasgow Alumni cohort is a historical cohort of students attending the University of Glasgow between 1948 and 1968 a time period which precedes the documented rise in asthma. Therefore the Glasgow Alumni Cohort uniquely offers the opportunity to investigate the association between socioeconomic position and asthma prior to the rise in asthma.

## Methods

### Study participants

The Glasgow Alumni Cohort has been described in detail elsewhere [15]. Briefly students attending Glasgow University from 1948 to 1968 were invited to attend a health examination at the Student Health Service. Medical history, sociodemographic and socioeconomic characteristics and physical examination were recorded through a physician-administered standardised questionnaire. Medical history included conditions such as bronchitis, asthma, hay fever, eczema/urticaria and questions on health behaviours. Data was collected on 15,322 students of which 11,756 were men, representing approximately 50 % of the student population. Students who were more than 30 years old at the time of examination were excluded (n = 482) leaving 11,274 participants.

Since 1998, 82.5 % of the total Glasgow Alumni Cohort has been successfully traced through the National Health Service Central Register. Between 2001 and 2002, members of the cohort that had been traced were asked to complete a postal questionnaire to collect additional early life and adulthood information and respiratory and other chronic diseases. Completed questionnaires were returned by 4,101 participants. Ethics committee approval was received for the study.

### Variable definitions

The early life outcome variables included were previous medical history of asthma, eczema/urticaria, hay fever and bronchitis measured at University entry through a physician-administered standardised questionnaire. These were categorized as asthma alone (asthma without eczema/urticaria or hay fever), asthma with atopy (asthma with eczema/urticaria and/or hay fever), and atopy alone (eczema/urticaria and/or hay fever without asthma). Eczema and urticaria were asked jointly in the questionnaire and could not be separated in the analysis. To evaluate whether this had an effect on the socioeconomic patterning of atopy, additional analysis were carried out defining atopy as reporting of hay fever only. Asthma ascertained in the adult postal follow-up was categorized as asthma ever (doctor diagnosis of asthma ever) and adult-onset asthma (doctor diagnosis of asthma ever combined with age of diagnosis greater than 30 years of age). Bronchitis was ascertained in the adult postal questionnaire if the participant responded positively to the question ‘Have you ever been told by a doctor that you have, or have had, bronchitis?’

Early life SEP was based on father’s occupational class (collected at university entry questionnaire) and classified on a five point scale from I (professional occupation), II (managerial and technical job), III (skilled non-manual and manual job), VI (partly skilled manual job) and V (unskilled manual jobs) using the Registrar General’s Classification, the official UK social class classification at that time (and until 2002) [16]. Due to small numbers social class groups III, IV and V were combined into one single group. The postal adult follow-up questionnaire included recall of early life SEP based on father’s main occupation, which was classified following the same standardized criteria into I–V groups, number of household amenities when starting university (included fridge, telephone, car and television), adult SEP based on participants main occupation (I–V), adult household crowding, income, home ownership and car ownership.

Other variables collected at University entry included birth order, number of siblings, smoking status and family history in first degree relatives of asthma, hay fever and eczema. Body Mass Index (BMI) was calculated from height and weight measured at University entry and used divided into tertiles to form a categorical variable as there were very few participants in the BMI > 30 when using WHO classification.

### Statistical analysis

Statistical analysis was completed in Stata 12.1 (Texas, USA). Chi-squared test was used to describe the association between (1) early life SEP and respiratory and atopic diseases and, (2) adult and early life SEP (as reported from the postal follow-up questionnaire) with adult respiratory diseases. We present the *p* value for trend using the exposure as a continuous variable. Multinomial logistic regression modelling was performed to determine the association between early life SEP and a 4-group outcome variable including asthma only, atopy only, asthma with atopy, and no asthma or atopy as the reference category. Results from these models are presented as multinomial odds ratios (MOR) [17, 18] although they are often referred to as relative risk ratios in the literature [19]. Multinomial logistic regression models were first adjusted for age and year of survey to investigate possible confounding by age of participant or change of definitions over time of surveys. Likelihood ratio tests (LRT) were used to test whether additional variables contributed to the model. The covariates considered for the adjusted model were; BMI, birth order, number of siblings and smoking status. The MOR and 95 % confidence intervals for the linear trend was determined by entering early life SEP as a continuous variable into the multinomial logistic regression model. Logistic regression models were used to investigate whether the association between asthma and early life SEP differed according to atopy status. The interaction was formally evaluated through a LRT comparing models with and without the interaction term. Logistic regression models were used to investigate the association between early life SEP and early life asthma classifying the latter as asthma yes/no (ignoring atopy status) and equal to adult asthma.

Finally, logistic regression modelling was performed to determine the association between early life SEP and adult onset asthma and asthma ever using data from the adult follow-up.

## Results

The age and survey year adjusted prevalence of asthma only at university entry was 2.38 % (95 % confidence interval (CI) 2.10–2.66), 1.25 % (95 % CI 1.04–1.46) for asthma with atopy and 6.52 % (95 % CI 6.06–6.97) for atopy only (eczema/urticaria and/or hay fever but no asthma). The age and survey year adjusted prevalence of bronchitis was 7.36 % (95 % CI 6.87–7.83).

Students who reported asthma only were younger, were from a lower socioeconomic background and had lower BMI (Table 1). Students categorised as asthmatics and atopics tended to be first born, report a family history of asthma and eczema and/or hay fever and from a higher SEP (Table 1). When using father’s social class as a four category variable the results for asthma with atopy were 2.05, 1.35, 0.73 and 0.91 % (*p*_trend_ < 0.001) for SEP groups I, II III, IV + V respectively and for asthma alone 1.92, 2.04, 2.83, 2.48 % (*p*_trend_ = 0.02) for SEP groups I, II III, IV + V respectively. Students who reported atopy only (eczema/urticaria and/or hay fever only) were younger, were more likely to be first born, have fewer siblings, report a family history of asthma and eczema and/or hay fever and be from a higher SEP. Those students who reported a medical history of bronchitis were also thinner, more likely to be first born, had fewer siblings, report a family history of asthma and eczema and/or hay fever and were more likely to have reported eczema/urticaria and/or hay fever (16.3 vs 7.2 % *p* < 0.001).

**Table 1:** Participants characteristics of male students in the Glasgow Alumni cohort (n = 11,274)

	No asthma or atopy (n = 10 130)	Asthma only (n = 268)	Asthma with atopy (n = 141)	Atopy only (n = 735)	*p* value	Bronchitis (n = 829)	
	N (%)	N (%)	N (%)	N (%)		N (%)	*p*_trend_
Age							
16–20	5,505 (89.2)	168 (2.72)	73 (1.18)	427 (6.92)	<0.001	432 (7.00)	0.21
21–25	3,656 (90.0)	81 (1.99)	62 (1.53)	264 (6.50)		320 (7.88)	
26–30	969 (93.4)	19 (1.83)	6 (0.58)	44 (4.24)		77 (7.42)	
Father’s social class							
I	1,923 (87.8)	42 (1.92)	45 (2.05)	180 (8.22)	<0.001	178 (8.13)	0.11
II	3,529 (90.2)	80 (2.04)	53 (1.35)	251 (6.41)		289 (7.39)	
III + IV + V	4,326 (90.8)	132 (2.77)	36 (0.76)	269 (5.65)		344 (7.07)	
BMI tertiles							
1	3,242 (89.2)	112 (3.08)	65 (1.79)	216 (5.94)	<0.001	324 (8.91)	0.001
2	3,502 (89.5)	91 (2.33)	38 (0.97)	280 (7.16)		248 (6.34)	
3	3,335 (90.9)	63 (1.72)	37 (1.01)	236 (6.43)		251 (6.84)	
Birth order							
1	5,368 (88.8)	157 (2.60)	90 (1.49)	430 (7.11)	0.001	478 (7.91)	0.001
2	2,700 (90.4)	68 (2.28)	31 (1.04)	187 (6.26)		209 (7.00)	
3+	1,939 (92.1)	41 (1.95)	15 (0.71)	111 (5.27)		133 (6.32)	
Number of siblings							
0	1,824 (88.0)	56 (2.70)	26 (1.25)	168 (8.10)	0.06	173 (8.34)	0.001
1	3,649 (89.9)	92 (2.27)	49 (1.21)	267 (6.58)		324 (7.99)	
2	2,418 (90.0)	64 (2.38)	38 (1.41)	168 (6.25)		176 (6.55)	
3+	2,235 (91.3)	56 (2.28)	28 (1.14)	132 (5.39)		155 (6.32)	
Smoking status							
Yes	6,348 (89.6)	84 (2.31)	33 (0.91)	463 (6.54)	0.12	292 (8.02)	0.05
No	3,286 (90.2)	172 (2.43)	101 (1.43)	240 (6.59)		495 (6.99)	
Family history of asthma							
Yes	394 (76.7)	37 (7.20)	26 (4.68)	55 (10.7)	<0.001	66 (12.0)	<0.001
No	9,736 (90.5)	231 (2.15)	113 (1.05)	680 (6.32)		763 (7.09)	
Family history of eczema and/or hay fever							
Yes	397 (71.5)	24 (4.32)	26 (4.69)	108 (19.5)	<0.001	57 (10.3)	<0.001
No	9,733 (90.8)	244 (2.28)	115 (1.07)	627 (5.85)		772 (7.20)	

In multinomial logistic regression modelling lower father’s occupational class was associated with higher odds of asthma only (trend aMOR = 1.22, 95 % CI 1.03–1.46) but a lower odds of asthma with atopy (asthma with eczema/urticaria or hay fever) (trend aMOR = 0.66, 95 % CI 0.51–0.81) compared to participants with no asthma or atopy and this relationship remain unchanged after adjusting for BMI, birth order, number of siblings, smoking status and family history of asthma and family history of eczema and/or hay fever (Table 2). Lower early life SEP was associated with a lower odds of atopy only (trend aMOR = 0.84, 95 % CI 0.75–0.93). These results were the same when we defined atopy with hay fever only, with the exception of the association of early life SEP with asthma alone that become non-statistically significant (trend aMOR = 1.08, 95 % CI 0.92–1.26). Asthma reported at university entry (classified as a binary variable ignoring atopy status) was not associated with father’s social class (OR_trend_ = 0.97, 95 % CI 0.85–1.11).

**Table 2:** The relationship between father’s social class and respiratory and allergic outcomes. Results presented as multinomial odds ratios (95 % confidence intervals)

	Social class			
	I	II	III + IV + V	Linear trend
No asthma or atopy			*Reference group*	
Asthma only				
Model 1	1	1.02 (0.69–1.50)	1.41 (0.98–2.02)	1.22 (1.03–1.46)
Model 2	1	1.03 (0.70–1.52)	1.42 (0.99–2.05)	1.23 (1.03–1.47)
Asthma with atopy				
Model 1	1	0.71 (0.46–1.08)	0.41 (0.25–0.66)	0.65 (0.51–0.81)
Model 2	1	0.72 (0.47–1.11)	0.43 (0.27–0.68)	0.66 (0.52–0.83)
Atopy only				
Model 1	1	0.77 (0.63–0.94)	0.66 (0.54–0.81)	0.82 (0.74–0.90)
Model 2	1	0.78 (0.64–0.96)	0.69 (0.56–0.85)	0.84 (0.75–0.93)

Model 1—year of survey and age

Model 2—Model 1 plus BMI, birth order, and number of siblings, smoking status and family history of asthma and family history of eczema and/or hay fever

In stratified analysis to test whether atopy modified the association between early life father’s occupational class and asthma, the odds of asthma among non-atopic students was higher with lower class (OR_trend_ = 1.22, 95 % CI 1.03–1.45), whereas among atopic students the odds of asthma was lower with lower occupational class (OR_trend_ = 0.81, 95 % CI 0.64–1.04) (interaction *p* value <0.001).

Compared to participants who completed the students’ health survey and were included in the analysis those who responded to the adult follow-up questionnaire were similar except for a smaller proportion of non-smokers (at university entry) and a higher proportion of students with fewer siblings.

From data collected from the follow-up questionnaire in adulthood, participants who reported asthma ever in adulthood were more likely to have reported asthma and bronchitis in early life (Table 3). Participants who reported adult-onset asthma were more likely to report fewer amenities (<3) in early life, report bronchitis in early life, and those who reported bronchitis in adulthood were more likely to have reported asthma and bronchitis in childhood (Table 3). There was no association between smoking reported in adulthood and asthma ever or adult-onset asthma but individuals who reported bronchitis ever were more likely to be former or current smokers.

**Table 3:** Descriptive characteristics and associations between SEP and adult asthma (onset > 30 years) for men (n = 4,101)

	Asthma ever (n = 333)		Adult onset asthma (n = 165)		Bronchitis ever (n = 358)	
	N (%)	*p*_trend_	N (%)	*p*_trend_	N (%)	*p*_trend_
*Early life SEP*						
Fathers social class						
I	62 (9.08)	0.39	21 (3.27)	0.27	64 (9.34)	0.72
II	114 (8.78)		56 (4.43)		111 (8.55)	
III + IV + V	146 (8.11)		77 (4.32)		170 (9.49)	
Household amenities						
Less than 3	188 (8.88)	0.45	103 (4.91)	0.01	213 (10.1)	0.05
Greater than 3	145 (8.19)		57 (3.26)		145 (8.20)	
Early life respiratory conditions						
Asthma						
No	227 (6.05)	<0.001	157 (4.22)	0.29	303 (8.08)	<0.001
Yes	106 (72.6)		3 (2.31)		55 (38.7)	
Bronchitis						
No	266 (7.34)	<0.001	142 (3.96)	0.03	250 (6.91)	<0.001
Yes	67 (24.5)		18 (6.82)		108 (39.4)	
*Adult SEP*						
Social class (main job)						
I	146 (9.09)	0.16	72 (4.52)	0.08	135 (8.40)	0.24
II	106 (8.17)		45 (3.53)		134 (10.3)	
III + IV + V	8 (5.67)		3 (2.14)		11 (7.86)	
Income						
Less than £19999	52 (9.09)	0.63	25 (4.44)	0.88	59 (10.4)	0.01
£20000–£49999	169 (8.57)		75 (3.94)		193 (9.80)	
£50000 or more	100 (8.36)		52 (4.40)		87 (7.29)	
Crowding						
Greater than 1.4	76 (8.81)	0.79	44 (5.13)	0.11	89 (10.4)	0.20
Less than 1.4	257 (8.52)		116 (3.89)		269 (8.92)	
Homeowner						
Owned outright	224 (8.27)	0.39	111 (4.14)	0.97	243 (8.99)	0.43
Mortgaged	92 (9.15)		41 (4.12)		93 (9.25)	
Rented	14 (9.15)		6 (4.11)		17 (11.4)	
Car ownership						
0–2 cars	225 (8.67)	0.81	109 (4.25)	0.74	241 (9.31)	0.68
3 or more cars	104 (8.43)		49 (4.01)		110 (8.90)	
Smoking status						
None	139 (8.10)	0.76	1,626 (95.9)	0.54	115 (6.71)	<0.001
Former	164 (9.34)		1,661 (95.5)		198 (11.3)	
Current	30 (7.39)		390 (97.3)		45 (11.1)	

There was good agreement between father’s social class recorded at university entry and longest father’s social class recalled in the adult follow-up questionnaire (kappa = 0.55, *p* < 0.001). Father’s occupational class, either recalled in adulthood or obtained at University entry was not associated with adult onset asthma. There were no association between adult household crowding, adult occupation, income and car ownership with asthma ever and adult onset asthma (onset > 30 years). A higher proportion of participants who reported a medical history of bronchitis had lower incomes in adulthood (Table 3).

Fewer household amenities in early life were associated with a higher risk of bronchitis ever (OR = 1.25, 95 % CI 1.00–1.56) however adjusting for age attenuated this association (aOR = 1.08, 95 % CI 0.85–1.39). Household amenities (<3), in early life was associated with higher risk of adult onset asthma (OR = 1.48, 95 % CI 1.07–2.05) and after adjusting for age the association attenuated (aOR = 1.27, 95 % CI 0.83–1.93).

## Discussion

### Main findings

In this historical cohort of male university students, we found that a different socioeconomic patterning was already present before the rises in the prevalence of asthma and atopy occurred in the UK. Asthma alone was higher in students from lower socioeconomic background whilst lower SEP was associated with lower odds of asthma with atopy and atopy alone. In addition, number of household amenities when starting university was associated with higher risk of adult onset asthma and bronchitis ever however this was attenuated when adjusting for age. Adult SEP was not associated with adult onset asthma.

### Comparisons with other studies and interpretation

The prevalence of asthma with atopy and asthma alone in this cohort was 1.25 and 2.38 % respectively and is comparable to asthma prevalence reported in the University of Wales between 1950 and 1954 [20]. Graham et al. [21] reported a higher prevalence of childhood asthma in higher social classes and a higher frequency of history of asthma in non-manual classes compared to manual classes were reported in a national study of childhood asthma [22]. However wheezing is reported to be lower in higher social classes in the Schoolchild Chest Health Survey (1966) [23] and no socioeconomic patterning was found in the 1958 birth cohort [24]. Asthma severity in childhood has been more consistently associated with lower childhood SEP [25, 26]. Conflicting results in the direction of association between early life SEP and asthma may be due to the inability to separate asthma with or without atopy phenotypes in previous studies. Pearce et al. [27] have suggested the importance of considering asthma with atopy and asthma alone separately in a bid to assess different aetiological factors (other than atopy) in the development of asthma. In this cross-sectional study although asthma and atopy may co-exist we are not able to determine if atopy is causally related to asthma. However, the different socioeconomic patterning of these two phenotypes of wheeze and asthma also points to different aetiological mechanisms each relating to environmental exposures and/or disease awareness that are differently distributed by socioeconomic groups.

There is only one study that has previously reported an association between early life SEP and asthma according to atopic status and from a period preceding the well-documented rise in asthma prevalence in Sweden [14]. Bråbäck et al. [14] investigated the associations of SEP and asthma in cohorts born between 1952 and 1977 and reported low SEP was associated with a higher risk of asthma without rhinitis across all cohorts. On the other hand, asthma with rhinitis, initially more common in higher social classes became more prevalent in the lower groups due to a faster rise among the latter. Non-atopic wheeze (as categorised by IgE status), but not atopic wheeze, has also been shown to have a higher prevalence in lower social classes (as categorised by head of household occupation) in the Health Survey for England (ages 11 and over) [28].

Similarly, there are a limited number of studies that have looked at the associations between early life SEP and hay fever and eczema within the similar time period as our cohort. Eczema, diagnosed by a school medical officer, and self-reported hay fever was higher in higher social classes in a cohort of children born in 1958 [24]. Parentally-reported eczema in childhood (age 5 or earlier) has also been associated with higher parental education in a later cohort born in 1970 [29].

On the other hand, a higher prevalence of asthma with atopy and atopic conditions may be due to over-reporting among students from higher SEP (or under-reporting among low SEP students) who may have had greater awareness of these conditions and greater access to medical services. Although this has been previously suggested [21, 30] whether this is the case has not been established and a report from the 1958 birth cohort suggested that the over-reporting of eczema was among low and not the higher SEP groups, therefore implying an even greater differential between socioeconomic groups [24].

Results from Bråbäck et al. [14] showed the steepest increase of asthma with rhinitis was seen in the lowest SEP group indicating that perhaps changes in exposures or awareness were greater in this group. If the role of SEP has changed over time, and cannot be explained by changes in disease awareness, suggests that exposures that lead to allergy and sensitisation may have become similar across all SEP and explain the inconsistency in associations in contemporary studies.

Our findings for the association between birth order with asthma and hay fever have been reported previously in this [31] and other studies [5, 32]. Both asthma with atopy and atopy alone, were more prevalent in lower birth orders (first or second born). Although a trend of lower prevalence with lower birth orders was also present for asthma alone the magnitude of differences was smaller and it could be due to chance. Thus, it is not clear whether the two asthma phenotypes have different associations with birth order in this cohort. We could not test a previously reported interaction between birth order and SEP [31] due to the small numbers and wide confidence intervals when classifying asthma according to the presence of other atopic conditions. Higher number of siblings, in the other hand, was only associated with lower prevalence of hay fever and bronchitis.

In our analysis we have used a 4-category outcome that included asthma only (asthma without eczema/urticaria or hay fever), asthma with atopy (asthma with eczema/urticaria and/or hay fever), and atopy only (eczema/urticaria and/or hay fever without asthma) compared to no asthma and no atopy. Multinomial models allow analyzing the three outcome groups simultaneously against the same reference level and allow testing for statistical significance accounting for the probabilities of all response categories. The group characterized by “asthma with atopy” is however difficult to interpret as it includes subjects for whom the asthma is related to their atopic status and asthma that simply coincides with atopy [33]. Many studies in this area have used different reference groups often making comparisons across studies difficult and the debate of which categories of asthma and atopy to use is on-going [33, 34]. Stratifying for atopy status allows to formally test whether the association between asthma and early life SEP differs across atopy groups [33].

Follow-up data from this cohort demonstrated no association with early adulthood or adult SEP with doctor’s diagnosis of ever asthma. However doctor’s diagnosis of adult-onset asthma and bronchitis were more prevalent among those living in households with fewer amenities during early adulthood. This was attenuated when adjusting for age. We could not differentiate asthma types in adulthood according to atopic status as there was no information collected on eczema/urticaria and hay fever in the adult follow-up postal questionnaire. Other investigators have reported a higher prevalence of adult asthma in lower adult socioeconomic groups [11, 35] but only a very small number of students were of mid-low adult social class in this cohort (90–95 % of students were of class I and II in adulthood).

### Strengths and limitations

A major strength of this study is the unique historical timing of the cohort, before the increase in asthma and atopic conditions, and before widespread use of antibiotics and immunisations. We could separate types of asthma according to the presence (or absence) of other atopy-associated conditions and measured early life SEP at the same time. Although medical history data was collected by physicians we cannot rule out misclassification of disease. The strong correlations between asthma and bronchitis highlight the difficulty to differentiate these diagnosis.

Diagnosis of asthma and bronchitis are likely to have overlapped. Indeed, bronchitis appeared to have mixed characteristics with asthma with or without atopy, namely higher prevalence in the leanest groups, lower birth orders, lower number of siblings and family history of asthma and family history of eczema and/or hay fever (family history of bronchitis was not available), suggesting that a degree of misclassification occurred between these two conditions. Atopy was also more frequently reported among students with bronchitis. On the other hand, in a previous report from this study we found a higher mortality in adulthood due to respiratory conditions among the students reporting asthma or bronchitis in early adulthood but only history of bronchitis was associated with higher mortality due to cardiovascular disease (as previously reported in the literature) suggesting some degree of ability to differentiate between these two conditions [36].

Members of this cohort were students at the University of Glasgow from 1948 to 1968 and therefore are a select group of individuals who could afford high education [15] with the majority of participants indicating father’s social class to be I, II and III. Most participants to the survey had professional occupations in their adult lives (90–95 % in class I and II). This is likely to have limited the socioeconomic variability in adult asthma and may limit the generalisability of these results. However, the associations reported with early life SEP and types of asthma are less likely to be confounded by adult socioeconomic circumstances. Thus, this relative homogeneity of adult socioeconomic circumstances becomes a strength when evaluating the association with early life SEP.

Only men are included in this report because of the small number of women attending university from 1948 to 1968. There were no associations between early adulthood SEP and asthma with atopy, asthma without atopy, hay fever only, eczema/urticaria only, in women in this cohort (results available from the authors) but we cannot rule out lack of power to detect this association in women. Ethnicity was not recorded because most of students attending university at that time would have been white. Similarly, potential relevant explanatory variables, such as parental smoking or access to health care, were not recorded and could not be evaluated.

Although a great proportion (82.5 %) of the Glasgow Alumni Cohort was traced through the National Health Service Central a smaller proportion responded to the follow-up questionnaire. Those who did not respond were more likely to be smokers though there were no other differences between responders and non-responders. Although this could present a potential bias to our results this would only be the case if the results in the non-response group demonstrated an opposite social patterning of asthma. Moreover we have previously reported that the association of early-life weight (reported in the Student questionnaire) and type-2 diabetes onset in adulthood (from follow-up questionnaire) was similar to another cohort with a greater response rate (90 %) [37].

## Conclusions

Lower SEP in early life was associated with a higher risk of asthma without atopy but a lower risk of hay fever and asthma with atopy among men in a cohort that preceded the 1960s rise in asthma prevalence in the UK. Adult onset asthma was associated with early life household amenities which was explained by age. Investigating the opposite patterns of association between SEP and asthma may highlight differences in environmental factors which are related differently to the development of allergy conditions from asthma without atopy. This needs further investigating in cohorts with detailed objective phenotypic and exposure data.
